# Second‐Generation (44‐Channel) Suprachoroidal Retinal Prosthesis: Surgical Stability and Safety During a 2‐Year Clinical Trial

**DOI:** 10.1111/ceo.14502

**Published:** 2025-01-31

**Authors:** Penelope J. Allen, Maria Kolic, Elizabeth K. Baglin, Samuel A. Titchener, Jessica Kvansakul, David A. X. Nayagam, Jonathan Yeoh, Robert J. Briggs, Joel Villalobos, Christopher E. Williams, Myra B. McGuinness, Chi D. Luu, Matthew A. Petoe, Carla J. Abbott, for the Bionics Institute and Centre for Eye Research Australia Retinal Prosthesis Consortium, Penelope J Allen, Maria Kolic, Elizabeth K. Baglin, Samuel A. Titchener, Jessica Kvansakul, David AX Nayagam, Jonathan Yeoh, Robert J Briggs, Joel Villalobos, Christopher E. Williams, Myra B. McGuinness, Chi D Luu, Matthew A. Petoe, Carla J. Abbott, Lauren N Ayton, Nick Barnes, Peter J. Blamey, Owen Burns, Robert G Buttery, Daniel WK Chiu, Rosie CH Dawkins, Stephanie B. Epp, Dean Johnson, Lewis Karapanos, William Kentler, Hugh J. McDermott, Ceara McGowan, Rodney E. Millard, Peter M. Seligman, Robert K. Shepherd, Nicholas C. Sinclair, Mohit N. Shivdasani, Patrick C. Thien, Ross Thomas, Janine G. Walker, Kiera A. Young

**Affiliations:** ^1^ Centre for Eye Research Australia Royal Victorian Eye and Ear Hospital Melbourne Victoria Australia; ^2^ Department of Surgery (Ophthalmology) University of Melbourne Melbourne Victoria Australia; ^3^ Vitreoretinal Unit Royal Victorian Eye and Ear Hospital Melbourne Victoria Australia; ^4^ Bionics Institute Melbourne Victoria Australia; ^5^ Medical Bionics Department University of Melbourne Melbourne Victoria Australia; ^6^ Department of Clinical Pathology University of Melbourne Melbourne Victoria Australia; ^7^ Department of Surgery (Otolaryngology) University of Melbourne Melbourne Victoria Australia; ^8^ Ear, Nose and Throat Unit Royal Victorian Eye and Ear Hospital Melbourne Victoria Australia; ^9^ Department of Biomedical Engineering University of Melbourne Melbourne Victoria Australia; ^10^ Melbourne School of Population and Global Health University of Melbourne Melbourne Victoria Australia

**Keywords:** adverse events, bionic eye, retinitis pigmentosa, suprachoroidal retinal prosthesis, surgical safety

## Abstract

**Background:**

To assess the safety and stability profile of the suprachoroidal retinal prosthesis (ScRP) in participants with retinitis pigmentosa (RP) for 2 years from implantation.

**Methods:**

Four participants, with advanced RP and bare‐light perception vision were enrolled in a prospective, single arm unmasked interventional clinical trial and unilaterally implanted with a 44‐channel ScRP (NCT03406416). Electrical stimulation commenced in the psychophysics laboratory prior to use in local environments. Outcome measures included serious adverse events, adverse events, implant stability and implant functionality to assess the safety and stability profile over 2.0–2.7 years.

**Results:**

Surgical procedures took 204–260 min and were uncomplicated. Postoperative recovery was uneventful. Imaging confirmed the device position under the macula and the absence of retinal trauma. There were no serious adverse events and the adverse events that occurred were mild. All electrodes were functional at surgery completion, and only 3% electrodes lost functionality by study end. There was minor array movement (translational and rotational) within the first 10–15 weeks only. The electrode to retina distance increased as expected with fibrous capsule development, but plateaued in three of four participants within 12 months. Retinal and choroidal thicknesses were consistent with the underlying retinal dystrophic disease.

**Conclusions:**

The ScRP can be safely implanted in the suprachoroidal space and has minimal long‐term impacts on the eye, with no SAEs and only slight array movement seen over 2.0–2.7 years. Hence, the findings indicate approach feasibility and further multicentre studies are warranted.

## 1 Introduction

Worldwide, several retinal prostheses have proceeded to clinical trials in human participants [1, 2, 3]. From a commercial perspective, the Vivani Medical (formerly Second Sight Medical Products) device the Argus II achieved United States Federal Drug Administration Humanitarian Device Exemption approval and Retina Implant AG's device Alpha AMS and Pixium Vision's device IRIS achieved CE mark for European commercial distribution. However, all three of these devices have now been withdrawn from the commercial market. The varying surgical approaches across groups attest to the lack of consensus regarding the value of proximity to the residual neuronal elements versus the comparative stability and simplicity of each approach. Vivani Medical and Pixium Vision developed an epiretinal approach [4, 5] with the electrode array tacked to the surface of the retina, whereas Retina Implant AG developed a subretinal procedure [6, 7] for placement of their electrode array. The Osaka research team working with Nidek developed an intra‐scleral surgical approach using a scleral flap [8] and our group, working with Bionic Vision Technologies, developed an array to slide into the suprachoroidal space [9]. The intra‐scleral and suprachoroidal approaches position stimulating electrodes further away from the target neurons of the inner retina, but the surgical techniques are straightforward and provide demonstrable device stability.

Our proof of concept study (NCT01603576) conducted in three participants with retinitis pigmentosa (RP) between 2012 and 2014 showed that suprachoroidal electrical stimulation could successfully evoke phosphene vision and that the surgical approach was safe and reproducible [10]. Light localisation improved in all three participants and two participants could navigate an obstacle course. The prototype device consisted of an electrode array composed of a silicone substrate connected by a helical cable to a percutaneous connector. Early in the postoperative period, all participants developed a combined suprachoroidal and subretinal haemorrhage that cleared without repercussions in two participants. The third participant developed a fibrous reaction in the far temporal periphery which did not affect device efficacy. Longitudinal impedance and optical coherence tomography (OCT) measurements, respectively, confirmed that all electrodes remained functional, and arrays remained stable for 18 months. The only serious adverse events (SAEs) were predicable infections associated with the percutaneous connector. An increase in electrode to retina (ER) distance over time was likely caused by high stimulation currents and pulse rates, triggering inflammation or continued fibrotic growth around the arrays [10, 11]. Hence, subsequent preclinical testing determined charge limits designed to prevent stimulation‐invoked increases in ER distance in a second‐generation device [12]. The charge limits determined were 250 nC (nanocoulombs) for a single electrode and 500 nC for paired electrodes [12, 13].

The second‐generation suprachoroidal retinal prosthesis (ScRP) was designed based on feedback from the prototype trial participants and combined an increased number of electrodes with a wider field of stimulation. The implanted components comprised of an array with 44 electrodes, dual behind‐the‐ear receiver‐stimulators, and external components included a camera mounted on glasses and a vision processing unit. Due to alteration in the mechanical characteristics of the electrode array, a preclinical chronic passive study [14] was undertaken to demonstrate the safety and stability of the new array prior to proceeding to a second‐generation fully implantable clinical trial. Separate second‐generation ScRP clinical trial reports have shown functional benefits over 2 years in participants with end‐stage RP [13, 15, 16]. The aim of this current report is to provide data on surgical outcomes and device stability during the second‐generation ScRP clinical trial, where participants have been using the device in their home environment for 2.0–2.7 years.

## 2 Methods

### 2.1 Participants

Four participants with advanced RP were enrolled in the second‐generation ScRP clinical trial (clinicaltrial.gov registration NCT03406416, site Centre for Eye Research Australia, 13 February 2018) to assess device safety and efficacy during 2018. The trial was approved by the Royal Victorian Eye and Ear Hospital Human Research Ethics committee (16/1266H) and conducted according to the tenets of the Declaration of Helsinki. Written informed consent was obtained from all participants after explanation of the nature and possible consequences of the study. Details regarding participant eligibility and screening procedures have been described previously [15].

### 2.2 Bionic Vision Technologies ScRP Device Design

**FIGURE 1:**
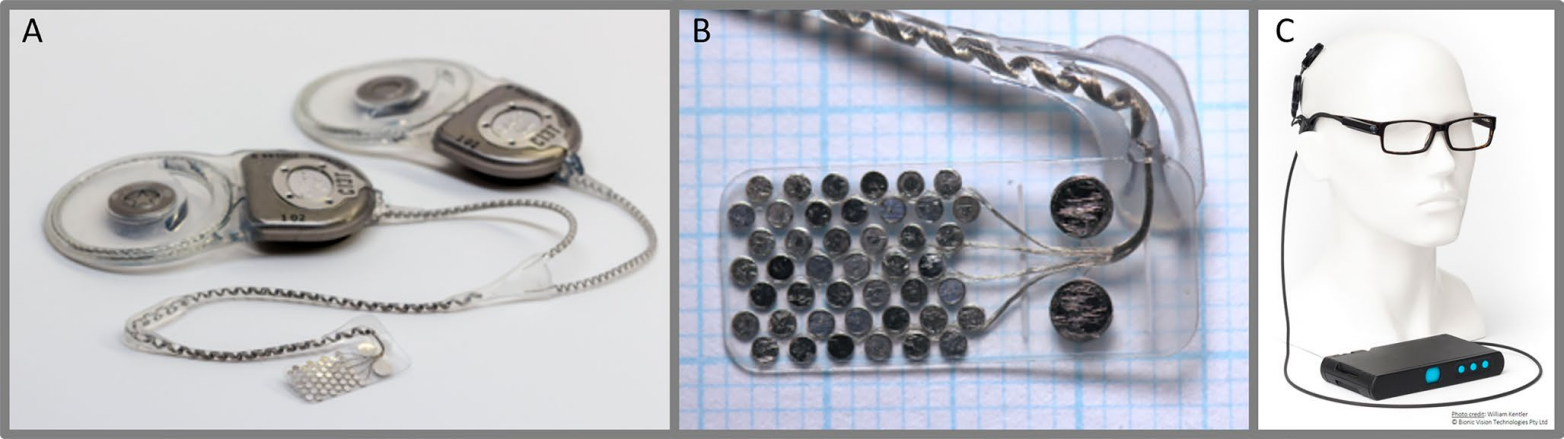
ScRP components. (A) Implantable device components, consisting of two magnetic coil receiver‐stimulators, each connecting to the electrode array via a cable with single strand helical wires from each electrode. (B) The electrode array, consisting of seven rows of 1 mm diameter platinum electrodes, with two larger return electrodes (2 mm diameter), imbedded in a rectangular silicone substrate, dimensions 19 ± 0.1 × 8 ± 0.1 × 0.6 mm (length, width, height). (C) External components of device for a right eye implant consisting of a small camera on the side of a pair of custom spectacles with a wired connection to the portable vision processing unit. Reprinted photographs under CC‐ND licences from Translational Vision Science and Technology, Vol 10 (10), Petoe et al., a second‐generation (44‐channel) suprachoroidal retinal prosthesis: Interim clinical trial results, p12, copyright (2021), and Translational Vision Science and Technology, Vol 11 (9), Abbott et al., inter‐observer agreement of electrode to retina distance measurement in a second‐generation (44‐channel) suprachoroidal retinal prosthesis, p4, copyright (2022).

The Bionic Vision Technologies second‐generation array is comprised of 44 platinum disc stimulating electrodes of 1 mm exposed diameter, and two platinum disc return electrodes of 2 mm exposed diameter (Figure 1). The electrodes are arranged in a staggered layout within the silicone substrate of size 19 × 8 mm. There is a cable with 46 helically coiled platinum wires exiting from the superior temporal corner, and a Dacron‐reinforced silicone patch attached to the cable for suturing to the sclera. Full details of the electrode array design has been shown previously [15]. In comparison to the prototype electrode array [9, 10], the silicone substrate has the same dimensions, however the diameter and number of electrodes has increased to both increase the field of view and to decrease the stimulation charge density.

Electrical stimulation was designed to be achieved by two current sources, packaged in two separate hermetically sealed titanium stimulator packages implanted under the postauricular scalp. Power and data transfer occur via head‐worn magnetically coupled transmission coils. The visual scene is continuously captured by a CMOS video camera mounted on the arm of a pair of spectacles and processed into suitable signals for the implanted stimulators by a body‐worn portable video processor.

### 2.3 Study Design

All participants received the retinal prosthesis unilaterally in their eye with their worse vision and hence were unmasked. The study was a single centre, single arm prospective interventional pilot study, with a recovery phase (Phase 1; 0–9 weeks post‐surgery), device fitting phase (Phase 2; switch‐on 7–9 weeks post‐surgery, fitting 0–16 weeks post switch‐on) and take‐home phase (Phase 3; 25‐weeks post switch‐on to study end). The device fitting phase incorporated initial psychophysics testing, including thresholding and determination of phosphene maps (defining ‘active’ electrodes, with electrodes excluded from the map being termed ‘passive’ electrodes), and camera training. The take‐home phase allowed participants to use their device outside the laboratory without supervision. Due to the impact of the COVID‐19 pandemic and the restrictions set by the Human Research Ethics Committee and hospital policy, the endpoint of the study was extended from the planned 2.0 years post switch‐on to up to 2.7 years. ‘Study end’ is defined as the last time where assessments could be achieved (Week 110 post switch‐on for S1, S2 and S3, and Week 140 post switch‐on for S4). All references to study weeks are relative to device switch‐on. The primary objective was to determine the safety of the device by recording device‐related SAEs. The secondary objective to assess efficacy of the device (functional vision) was presented separately [13, 16].

### 2.4 Surgical Procedure and Postoperative Care

**FIGURE 2:**
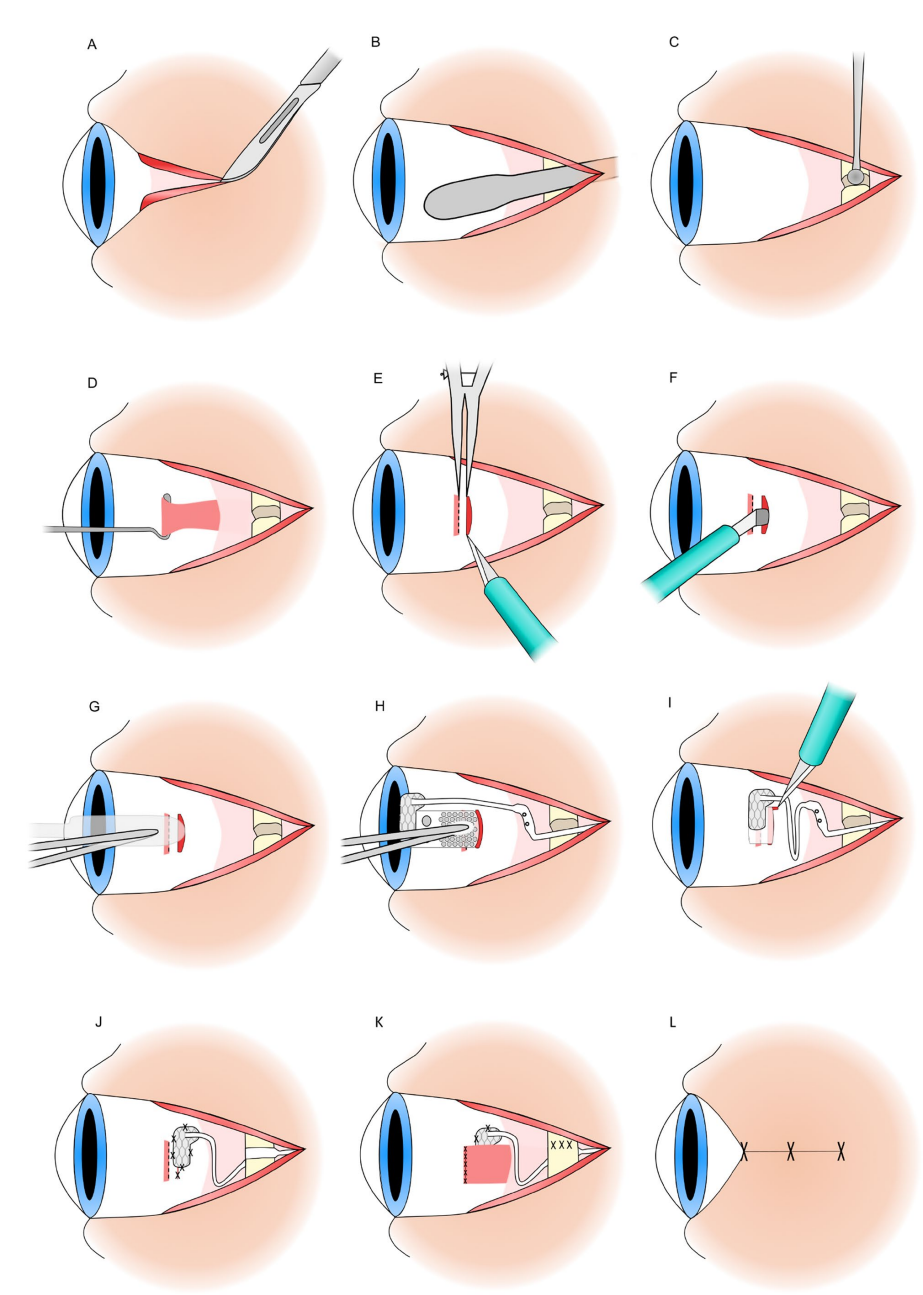
Surgical implantation of suprachoroidal device steps. (A) Lateral canthotomy is performed. (B) Dissection from wound behind pinna with trocar to enable passage of device. (C) Drilling of orbitotomy for cable stabilisation. (D) Isolation of lateral rectus muscle to temporarily disinsert it. (E) Creation of the scleral incision. (F) Dissection of suprachoroidal space. (G) Exploration of suprachoroidal space with lens glide. (H) Insertion of the electrode array into the suprachoroidal space beneath the macula. (I) Creation of L‐shaped extension of wound to allow egress of the lead. (J) The Dacron patch is sutured to the globe and the cable grommet is placed within the orbitotomy. (K) Lateral rectus is reattached, and the periosteum is closed over the cable. (L) All wounds are closed.

Devices were implanted by experienced vitreoretinal surgeons (P.J.A. and J.Y.) in collaboration with an otolaryngologist (R.J.B.) at the Royal Victorian Eye and Ear Hospital between February and August 2018. The surgical procedure has been previously described [9], and refined for the second‐generation ScRP device. General anaesthesia was administered followed by shaving the scalp around the intended site of the implantable receiver‐stimulators. Povidone iodine (Betadine) preparation was applied. The steps involved in the surgical procedure are summarised in Figure 2.

A dummy implant with a silicone cable was used for surgical planning and to prepare for a C‐shaped incision posterior to pinna. An incision was made through the temporalis muscle fascia to expose a flat section of squamous temporal bone for placement of the stimulator packages. The dummy implant was then used to confirm correct size and orientation when creating subperiosteal pockets and using a bone burr to drill wells in the temporal bone for the pedestal portion of the device. A tunnel was created beneath the temporalis muscle fascia forwards to the lateral orbital rim.

A lateral canthotomy was performed with the wound extended. The orbital margin was exposed, periosteum incised, and a flap created to expose the frontal process of the zygomatic bone. A lateral orbitotomy was created with a 1.0 mm burr to provide a notch for cable stabilisation. At this stage, the intraocular electrode array and cable, covered by Teflon, were loaded into a custom stainless‐steel trocar and passed forward from the postauricular incision, under the temporalis fascia and lateral to muscle, to the lateral canthotomy where the electrode array was unloaded. The receiver‐stimulators were placed within the preprepared wells in the temporal bone and the fascia closed over them. The cable was adjusted under the temporalis fascia with excess cable looped under the temporalis muscle.

A temporal peritomy was performed to expose the sclera and lateral rectus muscle before the lateral rectus muscle was disinserted. After consideration of preoperatively measured axial length, the intended scleral wound position was marked with diathermy. A 9 mm scleral wound was made with 15° and crescent blades (Alcon #8065990002, #8065921501), and the suprachoroidal space was dissected with a crescent blade and lens glide (BVI Visitec #581001). The electrode array was then inserted into the dissected suprachoroidal pocket, and the superior wound edge was extended posteriorly in an L‐shape to allow repositioning as necessary and a V‐shape sclerectomy was made to facilitate the cable exit. After the wound was stabilised with 8/0 nylon (Ethilon), the position and integrity of the device was checked with fundus examination and electrical impedance testing. The wound closure was completed with 8/0 nylon (Ethilon) and the Dacron‐reinforced patch was sutured to the sclera with 8/0 nylon (Ethilon) to stabilise the cable exit. The lateral rectus muscle was replaced in position, using 6/0 polyglactin 910 (Vicryl). The conjunctiva was closed with 8/0 polyglactin 910 (Vicryl).

With the ocular procedure completed, the cable was fixated in the orbitotomy by a silicone grommet and the periosteum closed over this with 8/0 nylon (Ethilon). The lateral canthotomy was closed in layers and the postauricular skin incision was repaired in two layers. Subtenon's local anaesthesia was injected for short‐term pain relief for the eye before a pressure dressing was applied to the postauricular surgical site.

**FIGURE 3:**
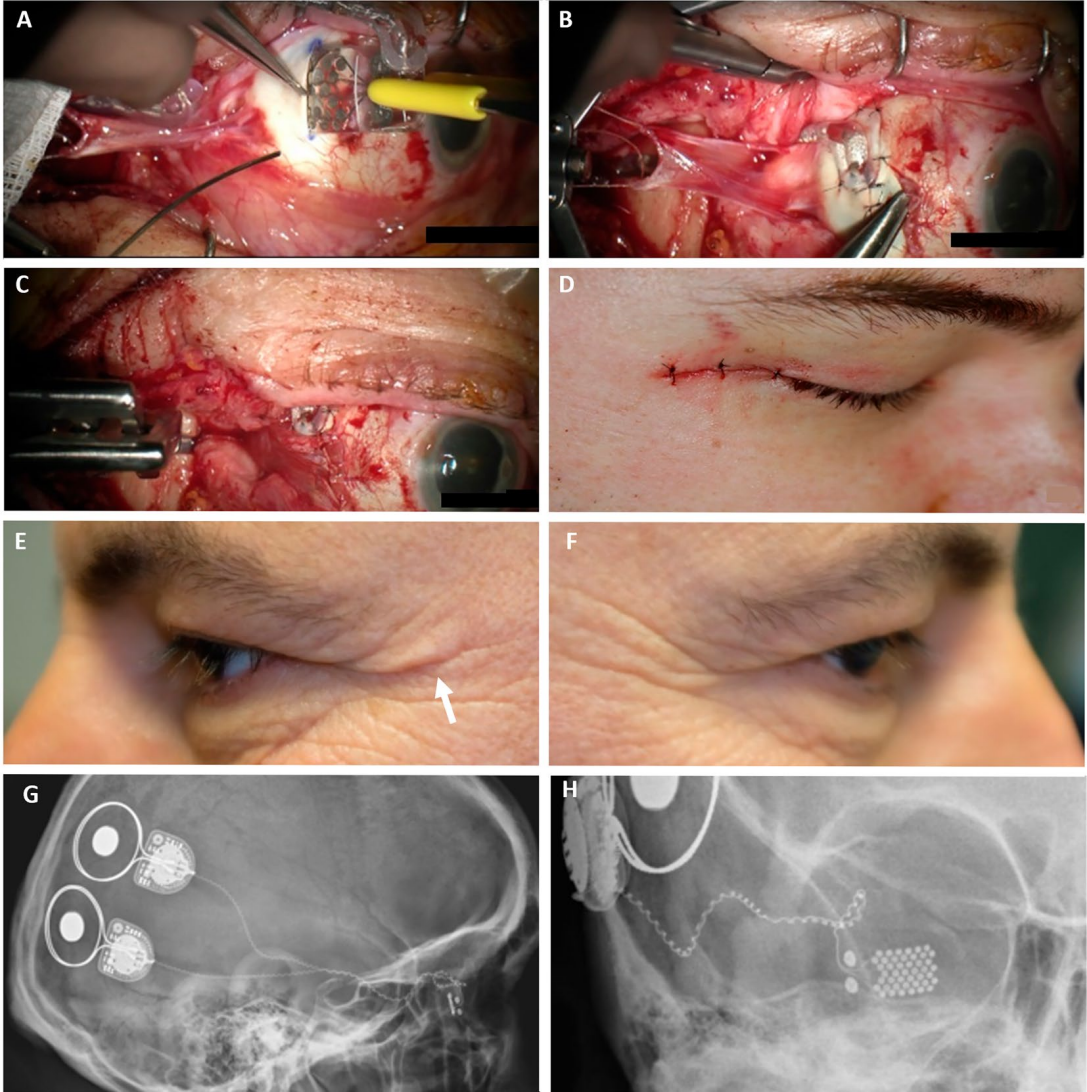
Photographs of key stages of the intraocular component of surgery and post‐surgical outcomes (S1) and X‐rays demonstrating the position of the implantable device components at 20 months post‐implantation (S4). (A) Electrode array being inserted into suprachoroidal space. (B) Suturing the Dacron patch. (C) Lateral rectus muscle reattached over the scleral wound. (D) Skin closure of lateral canthotomy. (E and F) Two‐year post‐implantation photographs demonstrating minimal scarring from the lateral canthotomy (arrow) in the implanted eye (E) compared to the fellow non‐implanted eye (F). (G) Lateral X‐ray of right side of skull showing the two receiver‐stimulators positioned on the temporal cranial bone. Each receiver‐stimulator is connected to the electrode array by the helical cable. (H) Anteroposterior X‐ray of right side of skull, with the eye in primary gaze, showing the suprachoroidal electrode array position and connection of the cable to the receiver‐stimulators.

Figure 3 shows intraoperative photographs of crucial steps in the procedure, including wound appearance at the completion of surgery and X‐ray images of one of the participants during the 2‐year follow‐up. Participants remained in hospital for 4 days. Intravenous antibiotics were administered for 48 h, followed by oral antibiotics for 5 days. Topical antibiotic chloramphenicol (chloramphenicol; Chlorsig) and steroid (prednisolone acetate, phenylephrine hydrochloride; Prednefrin forte) eye drops were administered regularly, with oral analgesia as required.

After discharge, eye examinations were performed initially weekly with a slit lamp, indirect ophthalmoscope, colour fundus photography and OCT to assess postoperative healing and ocular health. The external lateral canthotomy and postauricular wounds were monitored at each postoperative visit. X‐rays were obtained within the second year of the study.

### 2.5 Monitoring of Adverse Events

Safety data were collected from the date of surgical implantation and up to the study endpoint for each participant. All SAEs and adverse events (AEs) were documented and reported as per Good Clinical Practice guidelines (GCP/NHMRC/ICH E6). Subjective AEs were reported by the participant and objective AEs were reported by the principal investigator (P.J.A.). Event description, time of onset post‐procedure, severity, causality and outcomes were recorded and assessed by the principal investigator. The AEs related to the device were grouped into two categories: (i) after discharge but within 30 days from surgery, and (ii) from 30 days post‐surgery to study completion, to separate the AEs related to surgery from AEs related to the device presence and use. AE causality to device surgery, presence or use was classified as either not related, unlikely to be related, possibly related, probably related or definitely related. Only the AEs that were possibly, probably or definitely related to the device were included in analysis.

### 2.6 Device Stability

Colour fundus photography (TRC‐50EX, Topcon Medical Systems, Japan and Clarus 500, Carl Zeiss Meditec AG, Jena, Germany) and near infrared imaging plus OCT (Spectralis OCT, Heidelberg Engineering GmbH, Heidelberg, Germany) were conducted at least every 12 weeks. Near infrared images were montage stitched using the manufacturer provided Heyex software, then scaled and rotated to a common reference frame with Matlab 2020b (Mathworks, MA, USA). The known spacing between electrodes was used to convert pixels to millimetre. The location of the leading edge was inferred using the known geometry of the array. Quantification of longitudinal translational movement (mm) and rotational movement (°) of the leading edge of the array were calculated relative to baseline.

### 2.7 Device Functionality

Device functionality and hence the impact of the surgical insertion was assessed by electrical impedance testing of the electrodes at regular intervals using Custom Sound Suite software (Cochlear Australia, version 5.2) [17]. Initial impedance was performed at the end of surgery on the operating table to ensure the device had not been damaged during the surgery. Subsequently, impedance was performed regularly to document long‐term electrode functionality.

### 2.8 OCT Measurements

Retinal thickness (visualised in the manufacturer provided Heyex software as inner limiting membrane to inner boundary of the retinal pigment epithelium in these dystrophic retinae) was measured superficially to all electrodes that were able to be imaged at every time‐point. Measurements of choroidal thickness were taken overlying ‘active’ electrodes, ‘passive’ electrodes and over a similar region in the fellow eye shortly after implantation and at endpoint. The ER distance was measured for the entirety of the study using the method developed during our prototype study [10] that has been shown to be reliable [18].

## 3 Results

Participant characteristics were previously reported [13, 15] and are shown in Table S1. On genotyping, three of the four participants did not have a pathological mutation identified. All participants completed the study.

### 3.1 Adverse Events

All four participants recovered well after surgery, with no SAEs at any time during the study. Table 1 shows that all device‐related AEs within 30 days of surgery were mild to moderate and were primarily a prolongation of normal postoperative findings for comparable procedures. The presence of lid oedema was observed in all participants beyond the initial postoperative period, albeit this is not unusual in comparison to similar ocular procedures [19, 20], and settled spontaneously. Retinal haemorrhage as an adverse finding was expected after the prototype trial [10], however the degree of haemorrhage in the two affected participants in this study was milder and shorter lasting than reported in the prototype study, settling in 7–29 days with no sequelae.

**TABLE 1:** Reported adverse events (AE) classed as being related to the surgical implantation of the device up to 30 days post‐surgery.

Adverse event term	Number of participants reported AE	Severity	Duration mean (Days)	Duration range (Days)
Affect lability	1	Mild	1.00	1
Anterior chamber cell	1	Mild	1.00	1
Asthenopia	1	Mild	26.00	26
Conjunctival hyperemia	2	Mild	41.33	33–50
Discomfort—post auricular region	1	Mild	49.00	49
Extraocular discomfort	2	Mild	56.00	12–100
Foreign body sensation	1	Moderate	4.00	4
Headache	2	Mild–moderate	6.50	5–8
Intraocular pressure increase	1	Mild	6.00	6
Lethargy	1	Mild	8.00	8
Ocular discomfort	2	Mild	9.00	4–19
Retinal haemorrhage	2	Mild	18.00	7–29
Superficial pain—extraocular region	3	Mild–moderate	12.60	2–30
Superficial pain—post auricular region	3	Mild–moderate	5.33	5–6
Swelling of lid	4	Mild–moderate	53.50	33–75

**FIGURE 4:**
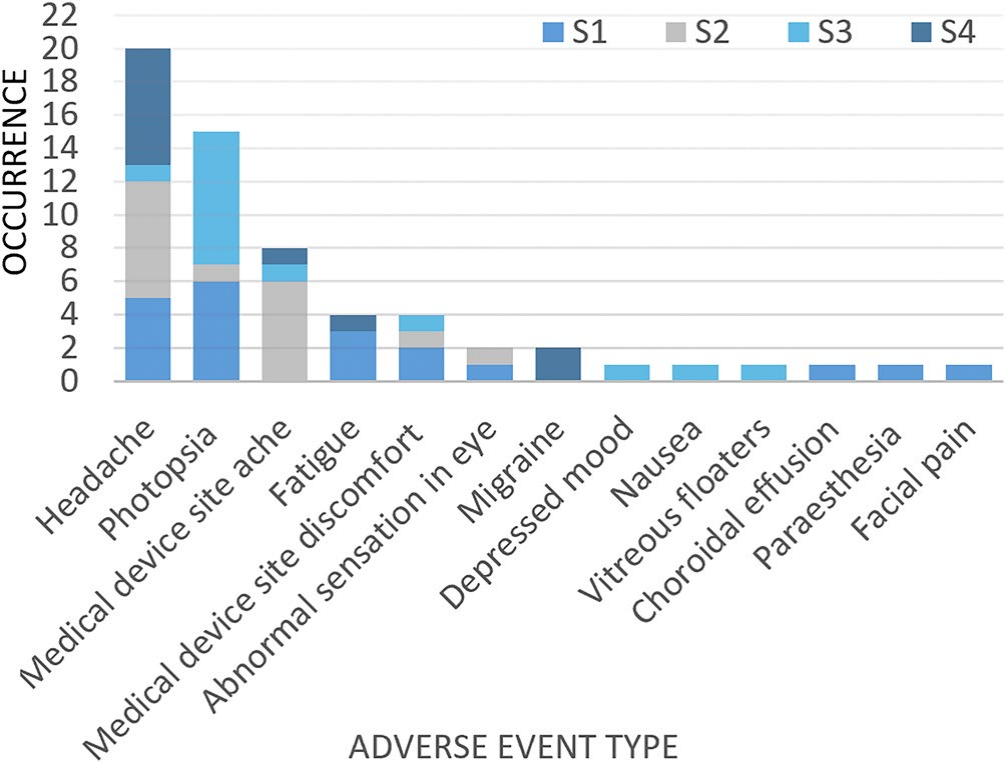
Occurrence of reported adverse events (AEs), from 30 days post‐surgical implantation to study endpoint. This graph includes reported AEs related to device use, study procedures and study tasks. AE events included are those that were classified as possibly, probably and definitely related to the device presence or use.

**FIGURE 5:**
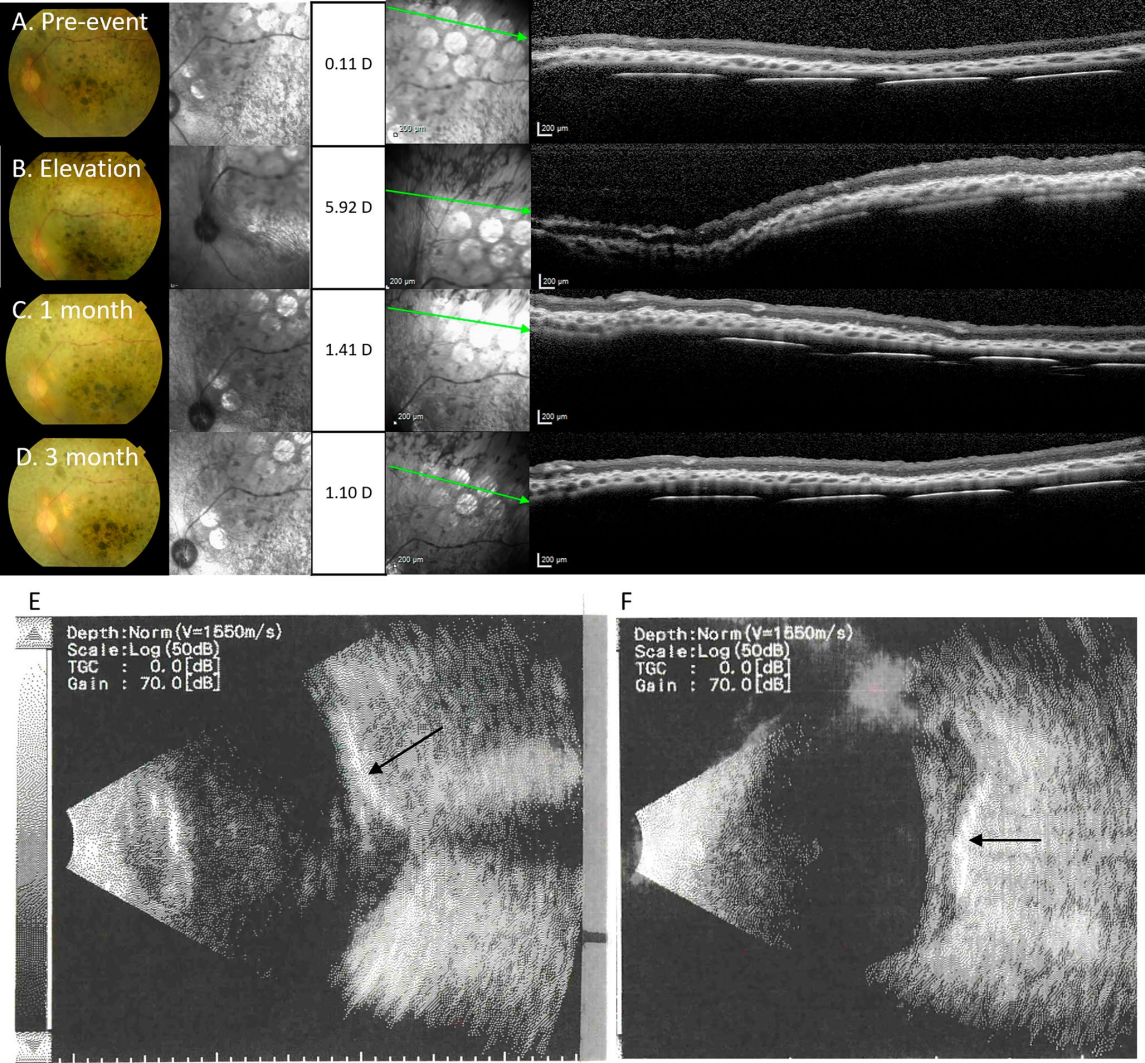
Choroidal effusion event in S1 noted at 2.5 months post‐implantation. (A–D) Colour fundus photos, near infrared images, the focus setting in Dioptres on the Heidelberg Spectralis and the OCT B‐scan image through the superior row of the electrode array, showing elevation of the array and overlying retina and the subsequent recovery over 3‐months ((A) at pre‐event, (B) retinal elevation during event, (C) 1‐month after event onset, (D) 3‐months after event onset). (E) B‐scan ultrasound positioned through optic nerve. (F) B‐scan ultrasound positioned temporal to optic nerve. Shadows posterior to the array are cast by the platinum electrodes. There was no sign of retinal, subretinal or suprachoroidal haemorrhage (no attenuation of ultrasound signal), hence the event was diagnosed as a choroidal effusion that resolved without treatment. Arrows indicate the position of electrode array within the suprachoroidal space.

Table 2 lists device‐related AEs occurring from 30 days post‐surgery and Figure 4 graphs these by frequency. The three most frequent AEs were headache, photopsia and device site ache, with only headache ever reaching a grading of severe. Two participants (S2 and S4) had one instance of severe headache classed as possibly or probably related to device use, noting they also had a history of severe headaches prior to trial enrolment. Generally, headache was fatigue associated and settled completely after the session finished. Photopsia after use of the device occurred in three participants and settled spontaneously. Device site ache settled completely within months of surgery and after this time palpation of the lead or stimulator site revealed no tenderness all the way through to the end of the study. The one AE of note is the development of a choroidal effusion in S1. This event began approximately 2.5‐months post‐surgery and had resolved by 5.5‐months post‐surgery (11 weeks in duration). S1 presented for routine review at 11 weeks post‐surgery with no new symptoms and no ocular pain. During OCT imaging, it was evident that the retina and array had ‘bulged forward’. Clinical examination showed a change in the contour of the device, however there was no evidence of haemorrhage, either subretinal or suprachoroidal. Figure 5 shows the colour fundus photograph, the near infrared image, the focus in Dioptres and the OCT B‐scan, demonstrating the longitudinal course of the event. B‐scan ultrasound (US‐4000 Nidek Echoscan) confirmed the change to the contour of the device and that there was no signal attenuation, consistent with choroidal effusion rather than haemorrhage. The time of onset of the event relative to surgery was longer than what would be usually expected for a post‐surgical effusion event. However, the effusion resolved and the device settled, returning to the pre‐event position. At no time did any haemorrhage become apparent either clinically or with imaging. Furthermore, there was no change in impedances, thresholds or functional vision. The event has not recurred in this participant or in any of the other participants.

**TABLE 2:** Reported adverse events (AE) classed as being related to the device presence or use from 30 days after surgical implantation until study endpoint.

Adverse event term	Number of participants reported AE	Severity	Duration mean (Days)	Duration range (Days)
Choroidal effusion	1	Moderate	78.00	78
Conjunctival hyperemia	1	Mild	7.00	7
Depressed mood	1	Mild	11.00	11
Discomfort—extraocular region	1	Mild	1.82	1–9
Discomfort—post auricular region	3	Mild–moderate	2.20	1–6
Discomfort—post auricular region (intermittent)	1	Mild	24.00	3–49
Dry eye	2	Mild	1.00	1
Fatigue	2	Mild	3.00	1–7
Headache	3	Mild–severe	1.00	1
Migraine	1	Mild	1.00	1
Nausea	1	Mild	1.00	1
Ocular discomfort	1	Mild–moderate	1.00	1
Pain—back	1	Moderate	6.00	6
Pain extraocular region	1	Mild	1.00	1
Pain—post auricular region	2	Mild	1.00	1
Paresthesia—temporal extraocular region	1	Mild	326.00	326
Photopsia—intermittent	3	Mild–moderate	107.87	1–756
Vitreous floaters	1	Mild	28.00	28

### 3.2 Device Stability

**FIGURE 6:**
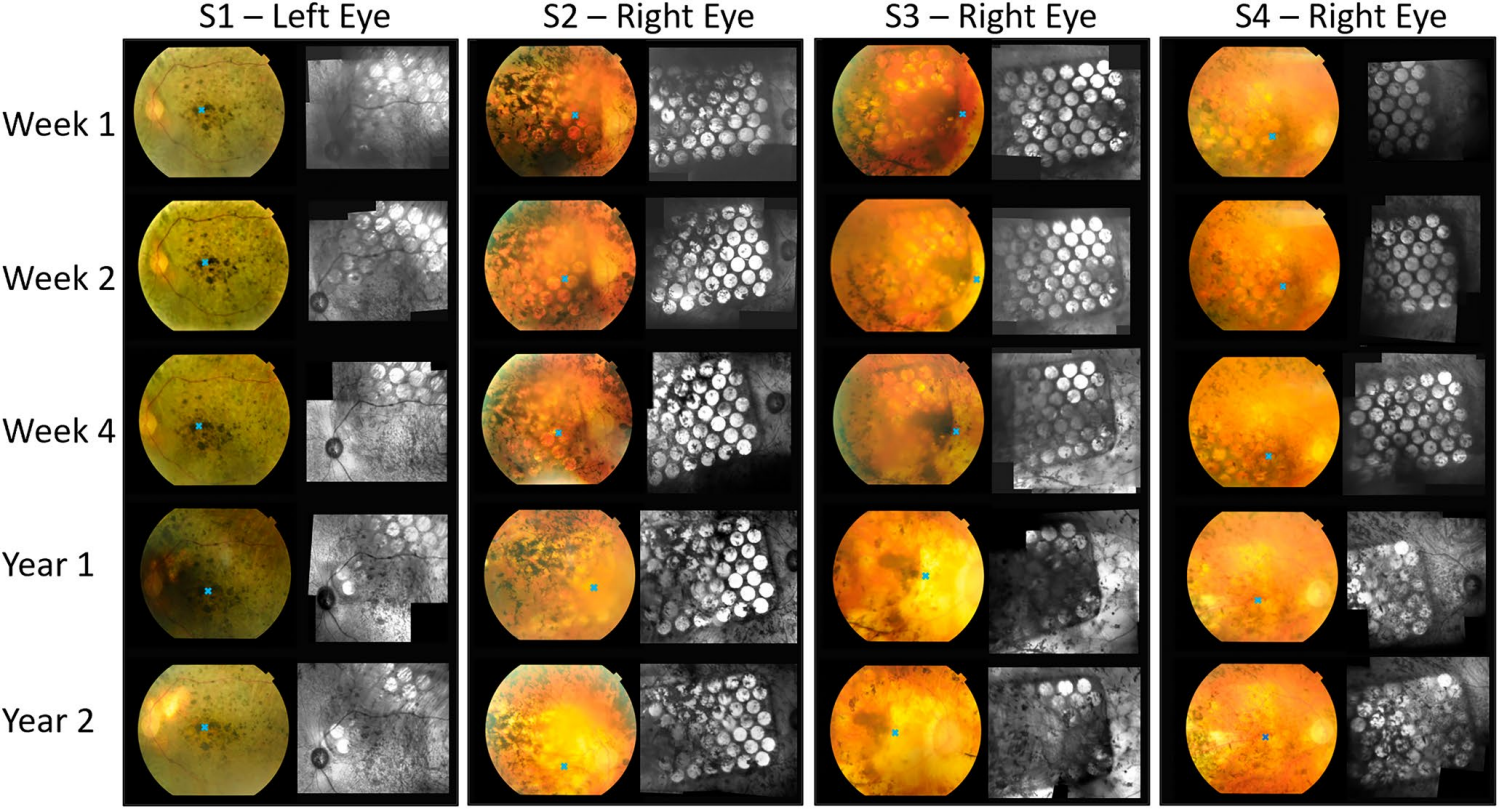
Colour fundus photos and near infrared images (montage stitched in Heyex software) showing the electrode array position for each participant over time. The foveal position is shown with a blue cross. All participants recovered well post‐surgery and the device remained stable within the suprachoroidal space for over 2 years. In S2 and S3, there were subtle subretinal haemorrhages that had resolved in 2 weeks. S1 had greater pigmentation of the ocular fundus due to racial variation that did not allow for viewing of the electrode array on colour fundus photo, however the array is visible on near infrared.

Figure 6 depicts the colour fundus photographs and near infrared images for all four participants demonstrating good array stability relative to the fovea over time. The subtle post‐surgical subretinal haemorrhages associated with the inferonasal end of the array are seen in S2 and S3. S1 has a more pigmented fundus due to racial variation, hence the device is more easily seen in the infrared images.

Representative (S4) plain X‐rays show the receiver‐stimulator units in the lateral X‐ray (Figure 3G) positioned postauricular with the cable from each unit meeting at a juncture, which was positioned in a groove in the skull. From that point, a single helical cable connects to the intraocular array. The anteroposterior film (Figure 3H) demonstrates the position of the intraocular array in primary gaze with a gentle loop of the intra‐orbital cable to the position of stabilisation at the frontal process of the zygomatic bone. X‐rays of all four participants confirmed the stability of the grommet and cable in the zygomatic bone and the smooth configuration of the intra‐orbital cable loop. Participants underwent regular testing of ocular motility showing no macroscopic limitation of ocular movements despite the two stabilisation points. However, some dampening of saccadic movement in the implanted eyes was observed with a high‐speed camera [21].

**FIGURE 7:**
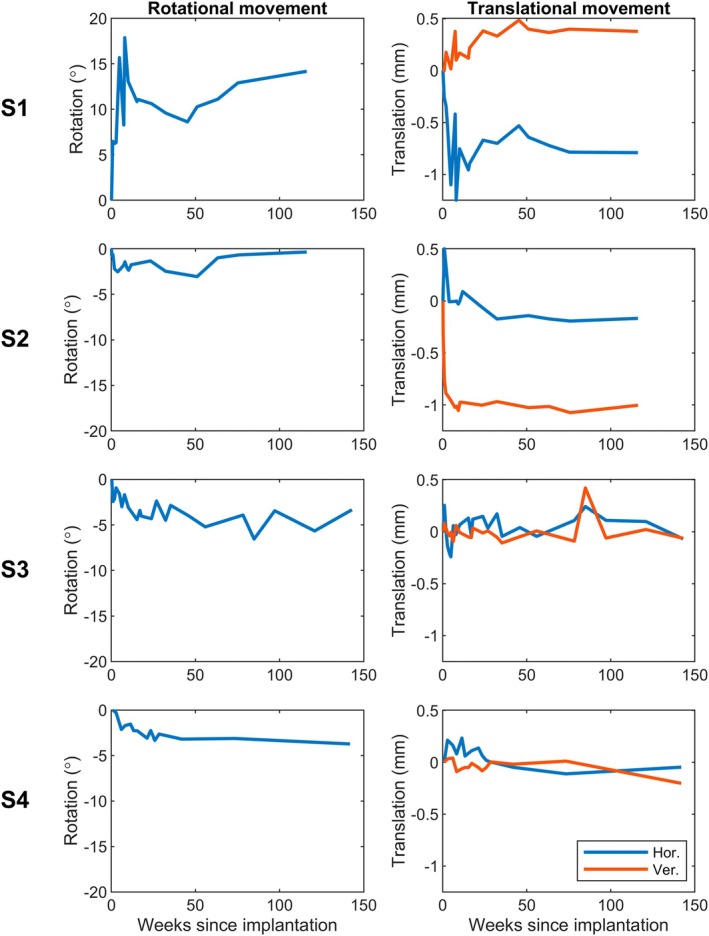
Rotational and translational movement of the electrode array in each participant relative to baseline. The translational movement was calculated for horizontal (Hor.) and vertical (Ver.) directions. The array is stable over time across participants after an initial settling period of 10–15 weeks.

Stability of neuro prostheses is very important for reliable long‐term stimulation. Figure 7 demonstrates the rotational and translational movement results, showing the array appears stable after an initial settling period, with greater initial rotation in S1 than the others. All participants have mild, early translational movement with stability being achieved after 10–15 weeks. Only S3 had a mild vertical change at about Week 100, which subsequently settled. The results show good stability of the leading edge over horizontal and vertical directions in three of the four participants (Figure S1), with only S1 demonstrating significant movement at the time of the presumed choroidal effusion.

### 3.3 Device Functionality

Detailed impedance data are provided in a separate publication [11]. The impedances increased slightly after surgery due to expected fluid onset, prior to plateauing after fluid clearance with the formation of a thin fibrotic capsule (Figure S2). All electrodes were functional intra‐operatively, with five electrodes in total (3%) becoming open‐circuit during the study. These electrodes were then disabled and excluded. The phosphene yield obtained within safe limits was 61.4% for S1; 72.7% for S2; 54.5% for S3 and 56.8% for S4, comprising predominantly foveal electrodes with sparser density at the periphery, and has been detailed previously [13, 15]. The phosphenes near the fovea were described by participants as having defined shapes, whereas those in the periphery were less defined ‘like sunrise peeping over the top of a hill’. The extent of retina subtended by the suprachoroidal electrodes (excluding return electrodes) is 38° × 28°; however, the maximum electrode eccentricity that produced a phosphene within the safety limits in the participants was 25° from the fovea. The field of view on average was 27.5° × 24.5° with all four participants being similar.

### 3.4 Retinal Measurements

**FIGURE 8:**
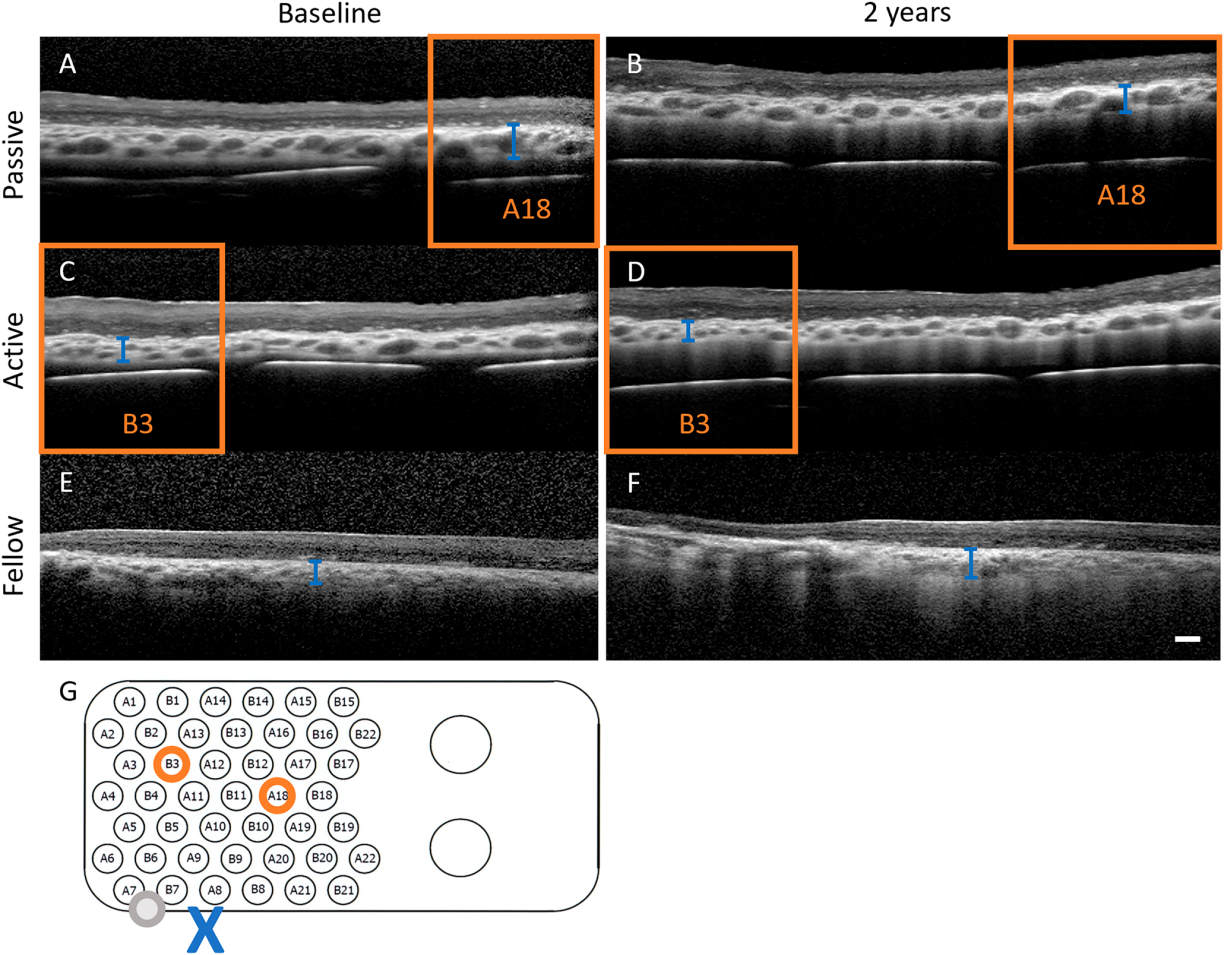
Representative OCT B‐scans from S1 showing the choroidal thickness remains consistent over 2 years above passive (A and B) and active (C and D) electrodes as well as in a comparative position (macula) of the fellow eye (E and F). The positions of the measurements are shown relative to the electrode array schematic (G). Orange box/circles indicates passive (A18) and active (B3) electrodes. Blue cross indicates the position of fovea relative to positions that choroid was measured (orange circles for implanted eye, grey circle for fellow eye). Blue line indicates choroidal thickness; passive baseline = 193 μm, passive 2 years = 195 μm; active baseline = 141 μm, active 2 years = 134 μm; fellow baseline = 212 μm, fellow 2 years = 204 μm. Scale bar = 200 μm.

Retinal thickness measurements were consistent with the underlying retinal dystrophic disease (Figure S3). The ER distance results show an early increase due to the expected development of a fibrous capsule around the suprachoroidal array, which stabilised by 12 months post‐surgery in three of four participants (S1, S2, S3; Figure S4) as previously reported [11]. In S4, the ER distance stabilises by the study end with a final ER distance similar to the other participants. Representative measurements of choroidal thickness (S1) are shown in Figure 8 and do not demonstrate any changes in over time or between ‘active’ and ‘passive’ electrodes or compared to a similar region in the non‐implanted fellow eye. The intraocular images indicate the electrode array is conformable to the shape of the eye, as the electrode array tip does not cause retinal folds or changes in pigmentation (Figure 6), the electrode tilt was minimal (Figures 8) and there were no SAEs.

## 4 Discussion

The key findings are that four participants underwent implantation of a second‐generation ScRP with no intraoperative complications and had fully functional devices at the completion of surgery with only five (3%) electrodes lost by study completion. The AEs were all expected, apart from the temporary choroidal effusion event in S1, which settled spontaneously with no repercussions. Measurements of device stability and retinal and choroidal thickness are further evidence of the potential of the ScRP approach and provide evidence of conformability.

Our approach leading to a safe human trial with a fully implantable device was built from a series of earlier studies. The suprachoroidal surgical procedure [9] was developed in a large eye animal model (feline) to demonstrate the feasibility and long‐term safety of suprachoroidal surgery and electrical stimulation prior to each clinical trial [14, 22, 23, 24]. Furthermore, given the prototype trial [10] showed a substantial increase in ER distance associated with higher current levels and rates, a second preclinical chronic study to clarify safe limits of electrical stimulation was performed prior to proceeding to this second‐generation ScRP clinical trial [12, 14]. The combined preclinical and prototype studies set up the success of the current clinical trial.

The straightforward surgical approach, the stability of the array in the suprachoroidal position and the larger coverage of visual field [15] seems to outweigh the disadvantage of the device being further from the retina. The results of this study, showing no SAEs over the study duration, confirm our initial findings from the prototype study [10] that placing the electrode array within the suprachoroidal space is surgically straightforward. As the procedure does not breach the vitreous cavity nor involve detaching the retina, it was unsurprising that the intraoperative complications were fewer and less severe than more complex procedures seen with other retinal implants [25, 26, 27, 28, 29], with the caveat that the suprachoroidal clinical trials currently have a small sample size. The arrays can be reliably placed beneath the macula and the design of the wound allows for final optimization of the array position, which can be checked visually in most patients. In patients with a more pigmented fundus, the use of an OCT integrated microscope would be useful, however this was not available at the time of these surgeries. This second‐generation device required the implantation of two receiver‐stimulators by otolaryngologist RGB which took approximately two‐thirds of the total operating time. The next generation device is planned to have a single receiver‐stimulator with no bony work required, significantly shortening surgical time.

The device‐related AEs documented in the initial 30 days after surgery are all consistent with ocular surgery of a similar nature, that is scleral wound repair or scleral buckling surgery [19, 20]. We anticipated that there would be suprachoroidal or subretinal haemorrhage in the early postoperative period after our experience in the prototype study [10]; however, this was minimal and resolved with no sequelae. The common device‐related AEs from 30 days post‐surgery to endpoint were expected and only one AE (headache) recorded a rating of severe in two instances. The one AE of note is the suprachoroidal effusion noted in S1. We obtained opinions from the members of the Vitreoretinal Unit at the Royal Victorian Eye and Ear Hospital (Melbourne, Australia) who either performed or observed the ultrasound at the time of the effusional event. All agreed that the B‐scan ultrasound was not consistent with haemorrhage but was consistent with effusion. We have been unable to explain the root cause, however, the spontaneous settling of the event and the consistency of functional responses despite the event is reassuring. Notably, we did not have any cases of endophthalmitis or severe intraocular inflammation, nor retinal detachment or hypotony, which are all SAEs noted by other retinal prosthesis groups [25, 26, 27, 28, 29].

Critically, this study showed minimal loss of electrode functionality despite the increased number of electrodes in the array and hence an increased number of wires in the cable compared to the prototype trial. These findings contrast with the device failure noted by Retina AG [7, 25, 30] and the loss of electrodes noted by Second Sight [26, 31]. The conformability of the array, the robust and compliant helical cable, and the stability of the array position within the eye explain this maintenance of functionality. The intraocular array is stable after some early minor movement and the cable, anchored at both the scleral exit and the orbital margin, remained stable. The position of the array did vary slightly between participants as shown in the fundus and near infrared images of Figure 6. This variance is greater than the rotation and translational movement documented in Figure 7, but interestingly had no correlation with performance, likely due to the large size of the array.

Assessment of retinal health in these participants with end‐stage dystrophic changes is clinically problematic. However, longitudinal measurements of retinal thickness showed only slow thinning consistent with the underlying disease and no oedema due to stimulation. The presence of the devices in the suprachoroidal space stimulated the development of a fibrous tissue capsule, but the ER distance also plateaued by 12 months and importantly did not lead to increased thresholds [11, 13].

The strengths of this study include documentation of all AEs during the 2 years of the study and the careful follow‐up of device position. The main limitation is our small cohort, however, publications from previous initial safety studies relating to new retinal prostheses have all had small cohorts. It is appropriate that the human research ethics committees are conservative when approving studies of new devices and operative approaches. We have continued to work with these participants post‐trial in an ongoing longitudinal study (#NCT05158049) to further document their progress regarding safety and stability monitoring and testing new vision processing algorithms to work towards improved functionality of the devices.

In conclusion, the safety profile and the surgical approach of the second‐generation ScRP is promising and the stability of the device in the suprachoroidal space is also pleasing with no evidence of SAEs with over 2 years of implantation and use in home environments. We believe this evidence in conjunction with our companion papers detailing the functional outcomes from the trial [13, 15, 16] and the paper detailing the outcomes from our prototype study [10] warrants further multicentre studies and potential commercialisation of the device.

## Conflicts of Interest

P.J.A., D.A.X.N., J.V., C.E.W. and M.A.P. hold patents in relation to this work. P.J.A., M.K., E.K.B., S.A.T., J.K., D.A.X.N., C.D.L., M.A.P. and C.J.A. received financial support for the work from Bionic Vision Technologies. M.K., E.K.B., M.A.P. and C.J.A. received travel funding from Bionic Vision Technologies to present this work at an international conference in 2019. J.Y., R.J.B. and M.B.M. declare no conflicts of interest.

## Supporting information


**Data S1.** This article contains additional online‐only material in Appendix A (Table S1), Appendix B (Figures S1–S4) and Appendix C (Bionics Institute and Centre for Eye Research Australia Retinal Prosthesis Consortium Members).

## Data Availability

The data that support the findings of this study are available from the corresponding author upon reasonable request.
