# Supplementary material for: Second‐Generation (44‐Channel) Suprachoroidal Retinal Prosthesis: Surgical Stability and Safety During a 2‐Year Clinical Trial
Source: Clin Exp Ophthalmol. 2025 Jan 31;53(5):529–41. doi: 10.1111/ceo.14502 (PMC12235112; doi:10.1111/ceo.14502)
Supplement: Supplementary file 1 — Data S1. This article contains additional online‐only material in Appendix A (Table S1), Appendix B (Figures S1–S4) and Appendix C (Bionics Institute and Centre for Eye Research Australia Retinal Prosthesis Consortium Members). [file CEO-53-529-s001.docx]

**SUPPLEMENTARY MATERIALS**

**APPENDIX A: Supplementary Table**

**Supplementary Table S1.** Participant characteristics and genotyping.

| **Characteristic** | **S1** | **S2** | **S3** | **S4** |
| --- | --- | --- | --- | --- |
| Gender | Male | Male | Female | Male |
| Age at implant (years) | 47 | 63 | 66 | 39 |
| Eye condition | Retinitis pigmentosa (Rod cone dystrophy) | Retinitis pigmentosa (Rod cone dystrophy) | Retinitis pigmentosa (Cone rod dystrophy) | Retinitis pigmentosa (Cone rod dystrophy) |
| Genotyping | Autosomal recessive.  Homozygous for deletion ADAM9. | Negative for explaining phenotype. | Negative for explaining phenotype.  Heterozygous for IFT81. | Negative for explaining phenotype.  Heterozygous for NMNAT1.  Heterozygous for PEX26. |
| Observed nystagmus | Mild | Intermittent | None | Mild |
| Visual acuity | Light perception both eyes | Light perception both eyes | Light perception both eyes | Light perception both eyes |
| Age when legally blind | 20 | 34 | 41 | 13 |
| Approximate years of useful form vision | 34 | 43 | 56 | 19 |
| Primary mobility aid | Cane | Cane | Guide Dog | Cane |
| Implanted eye | Left | Right | Right | Right |

**APPENDIX B: Supplementary Figures**


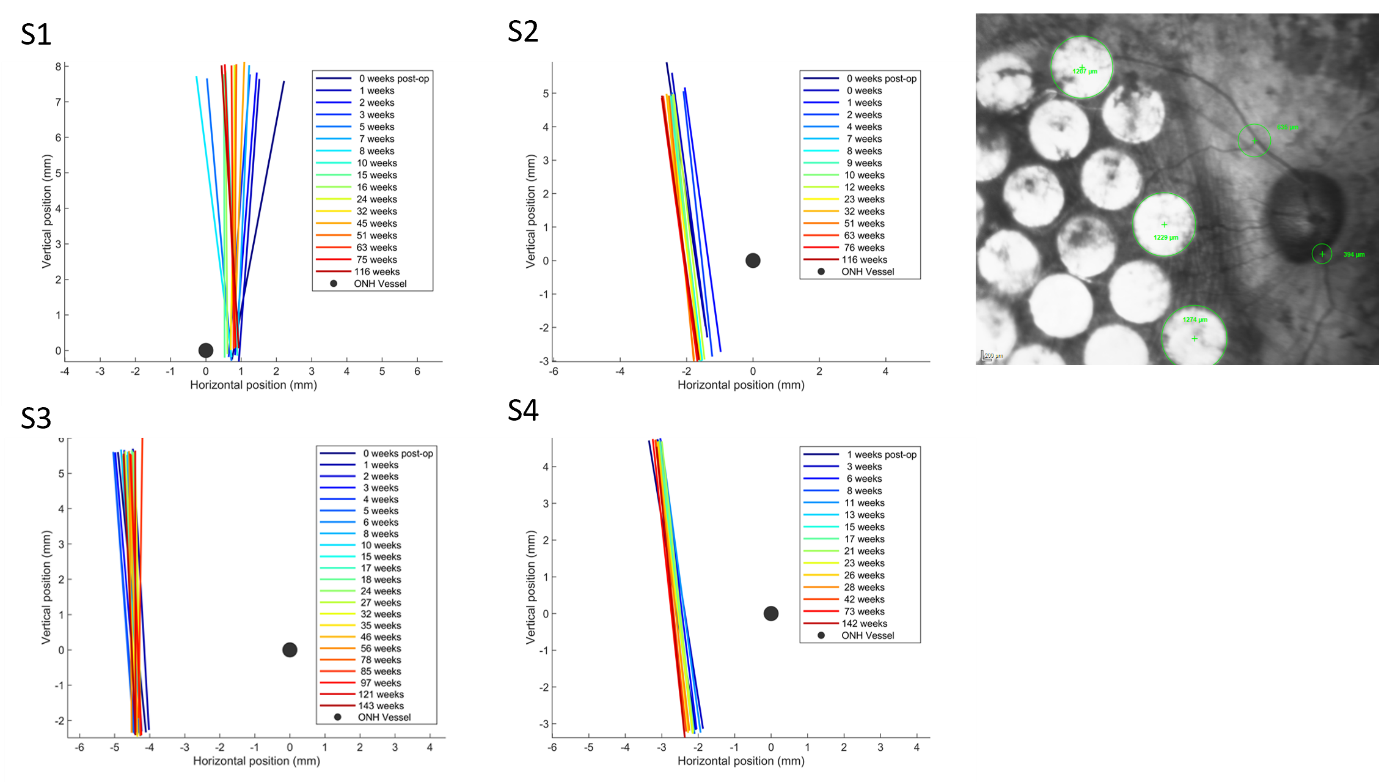


**Supplementary Figure S1.** Movement at the leading edge of the array relative to the optic nerve head over the study duration. The position of the leading edge is stable in three of four participants (S2, S3, S4) throughout the study. There is movement of the leading edge in one participant (S1), corresponding to the time of the presumed choroidal effusion event in this participant. The near infrared image depicts the method used to calculate the position of the array relative to retinal features and thus determine any movement that has occurred over time. The pixel locations of two prominent vascular features and the central location of 2 electrodes were marked. The relative angle of the vascular features was used to colocalize longitudinal images. The distance in pixels between the electrodes was compared to the known distance between them so the location (mm) and rotation (°) of the leading edge of the array relative to the optic nerve head could be determined.


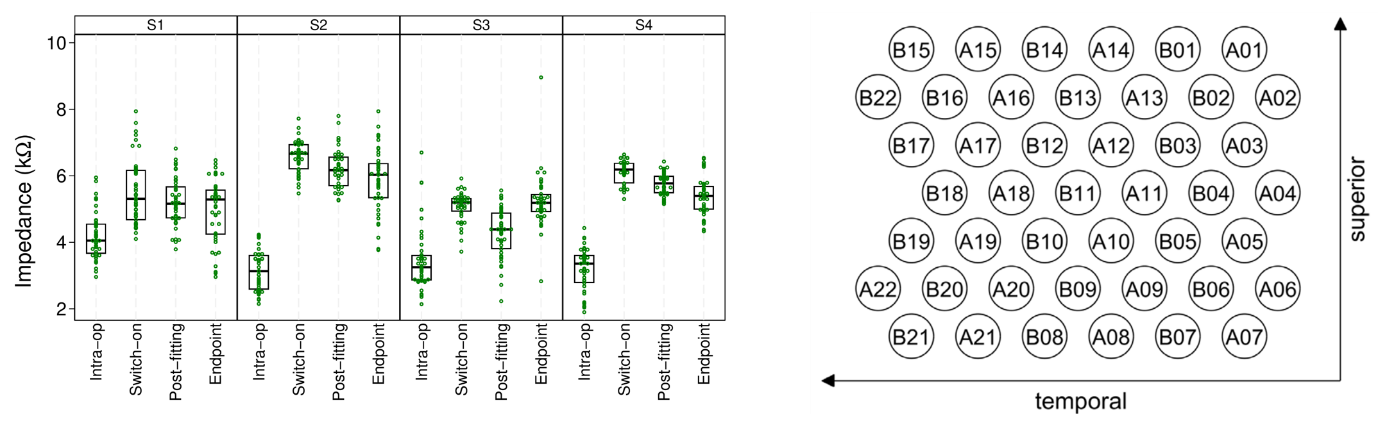


**Supplementary Figure S2.** Electrical impedance at four key timepoints: Intra-operation (intra-op), switch-on day (7-9 weeks post intra-op), immediately post-fitting (14-20 weeks post switch-on), and at study endpoint (2.0-2.7 years post switch-on). Each circle represents one electrode, box plots show the mean and interquartile range. Impedance increases by an expected amount from surgery to switch-on, and then remains stable over the study duration. N=43 electrodes/participant (excludes N=4 open circuit electrodes; S1=A07, S2=A10, S3=B01, S4=B22 as shown in the labelled array map).


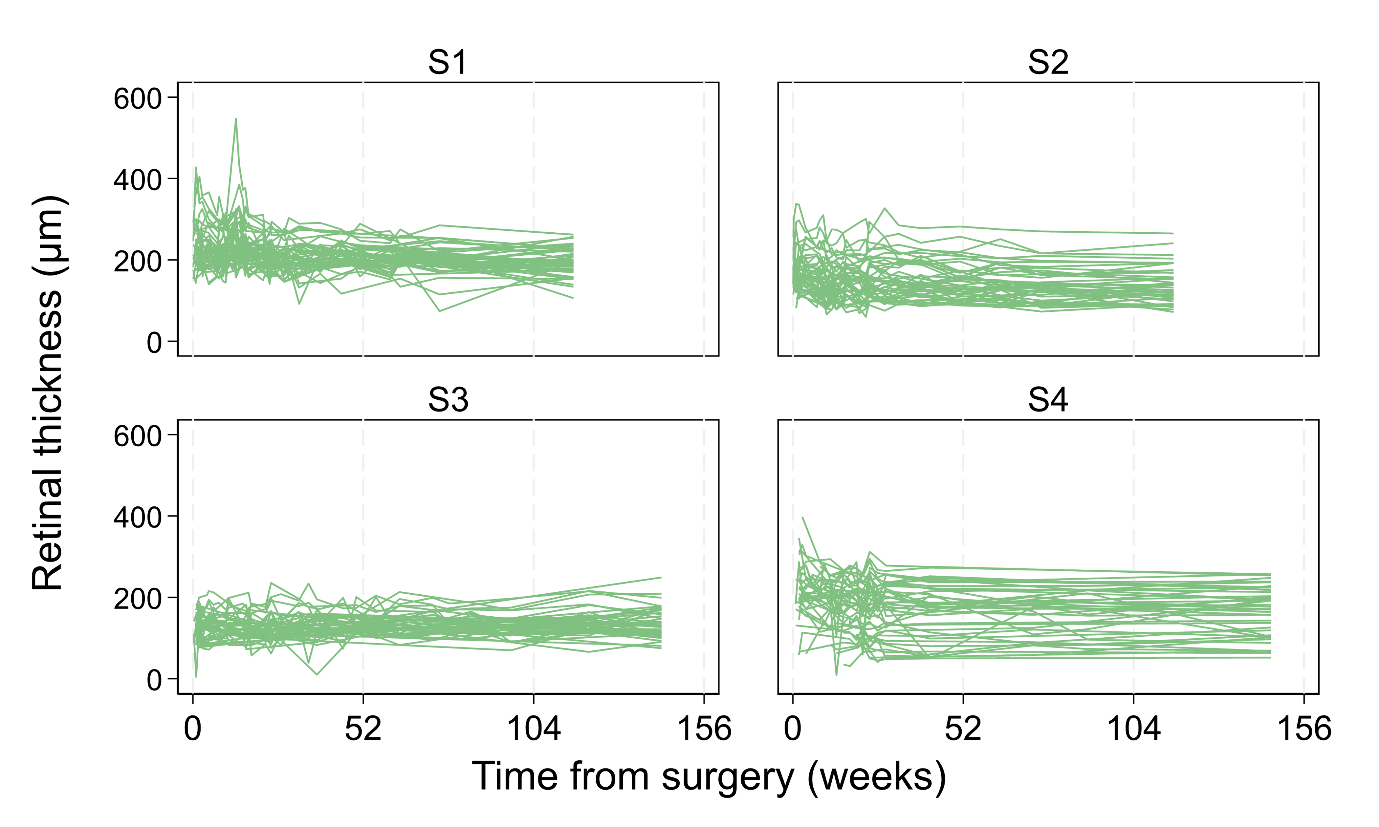


**Supplementary Figure S3**. Longitudinal retinal thickness measures for each participant from all measurable locations overlying the electrode array. There are minimal changes in retinal thickness over time, which is consistent with the loss of photoreceptors and retinal remodeling changes that are known to occur with retinitis pigmentosa.


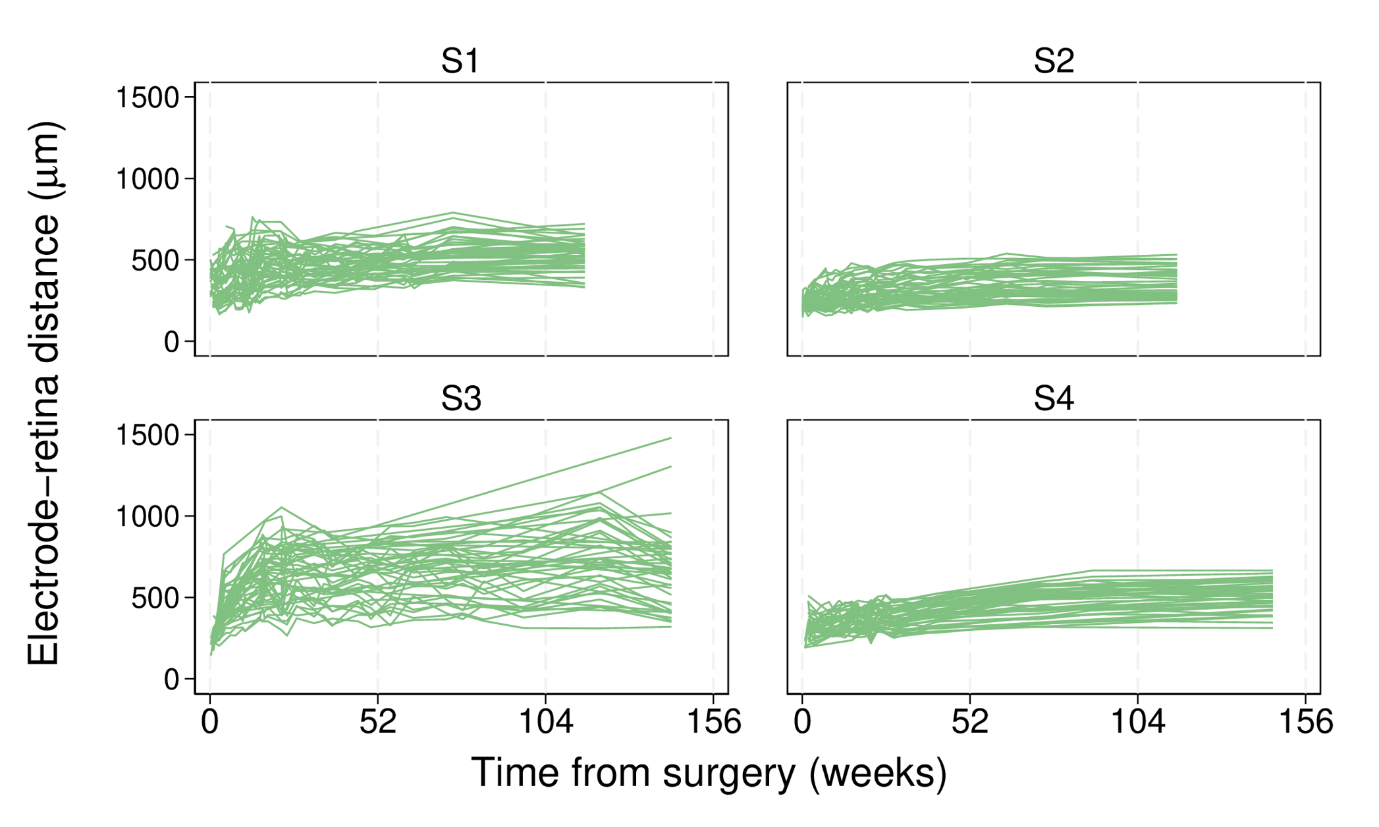


**Supplementary Figure S4.** Longitudinal electrode-retina (ER) distance measures for each electrode. ER distance increases initially after surgery, then stabilizes for S1, S2 and S3 at about 12 months post-surgery. For S4, the ER distance continues to increase but appears to stabilize by endpoint.

**APPENDIX C: Bionics Institute and Centre for Eye Research Australia Retinal Prosthesis Consortium Members**

**Writing Group Members:**

Penelope J Allen, Maria Kolic, Elizabeth K. Baglin, Samuel A. Titchener, Jessica Kvansakul, David AX Nayagam, Jonathan Yeoh, Robert J Briggs, Joel Villalobos, Christopher E. Williams, Myra B. McGuinness, Chi D Luu, Matthew A. Petoe and Carla J. Abbott

**Study Group Members:**

Lauren N Ayton; Nick Barnes; Peter J. Blamey; Owen Burns; Robert G Buttery; Daniel WK Chiu; Rosie CH Dawkins; Stephanie B. Epp; Dean Johnson; Lewis Karapanos; William Kentler; Hugh J. McDermott; Ceara McGowan; Rodney E. Millard; Peter M. Seligman; Robert K. Shepherd; Nicholas C. Sinclair; Mohit N. Shivdasani; Patrick C. Thien; Ross Thomas; Janine G. Walker; Kiera A. Young
